# Early Double-Negative Thymocyte Export in *Trypanosoma cruzi* Infection Is Restricted by Sphingosine Receptors and Associated with Human Chagas Disease

**DOI:** 10.1371/journal.pntd.0003203

**Published:** 2014-10-16

**Authors:** Ailin Lepletier, Liliane de Almeida, Leonardo Santos, Luzia da Silva Sampaio, Bruno Paredes, Florencia Belén González, Célio Geraldo Freire-de-Lima, Juan Beloscar, Oscar Bottasso, Marcelo Einicker-Lamas, Ana Rosa Pérez, Wilson Savino, Alexandre Morrot

**Affiliations:** 1 Laboratory on Thymus Research, Oswaldo Cruz Foundation, Rio de Janeiro, Brazil; 2 Institute of Microbiology, Federal University of Rio de Janeiro, Rio de Janeiro, Brazil; 3 Carlos Chagas Filho Institute of Biophysics, Federal University of Rio de Janeiro, Rio de Janeiro, Brazil; 4 Institute of Immunology, National University of Rosario, Rosario, Argentina; 5 Servicio de Clínica Médica, Hospital J.B. Iturraspe, Santa Fe, Argentina; Federal University of São Paulo, Brazil

## Abstract

The protozoan parasite *Trypanosoma cruzi* is able to target the thymus and induce alterations of the thymic microenvironmental and lymphoid compartments. Acute infection results in severe atrophy of the organ and early release of immature thymocytes into the periphery. To date, the pathophysiological effects of thymic changes promoted by parasite-inducing premature release of thymocytes to the periphery has remained elusive. Herein, we show that sphingosine-1-phosphate (S1P), a potent mediator of T cell chemotaxis, plays a role in the exit of immature double-negative thymocytes in experimental Chagas disease. In thymuses from *T. cruzi*-infected mice we detected reduced transcription of the S1P kinase 1 and 2 genes related to S1P biosynthesis, together with increased transcription of the SGPL1 sphingosine-1-lyase gene, whose product inactivates S1P. These changes were associated with reduced intrathymic levels of S1P kinase activity. Interestingly, double-negative thymocytes from infected animals expressed high levels of the S1P receptor during infection, and migrated to lower levels of S1P. Moreover, during *T. cruzi* infection, this thymocyte subset expresses high levels of IL-17 and TNF-α cytokines upon polyclonal stimulation. *In vivo* treatment with the S1P receptor antagonist FTY720 resulted in recovery the numbers of double-negative thymocytes in infected thymuses to physiological levels. Finally, we showed increased numbers of double-negative T cells in the peripheral blood in severe cardiac forms of human Chagas disease.

## Author Summary

The formation of mature lineage-committed T cells requires the specialized environment of the thymus, a central organ of the immune system supporting the development of self-tolerant T cells. Key events of intrathymic T-cell development include lineage commitment, selection events and thymic emigration. This organ undergoes physiological involution during aging. However, acute thymic atrophy can occur in the presence autoimmune diseases, malignant tumors and infections caused by intracellular pathogens. The present study shows that the protozoan parasite *Trypanosoma cruzi* changes the thymic microenvironmental and lymphoid compartments, resulting in premature release of very immature CD4^−^CD8^−^ double-negative thymocytes, TCRneg/low, which bear a pro-inflammatory activation profile. Strikingly, we also found elevated levels of these undifferentiated T lymphocytes in the peripheral blood of patients in severe cardiac forms of chronic Chagas disease. Importantly, we provided evidence that migration of CD4^−^CD8^−^ T cells from infected mouse thymus is due to sphingosine-1-phosphate receptor-1-dependent chemotaxis. These findings point to an important role for bioactive signaling sphingolipids in the thymic escape of immature thymocytes to the periphery in Chagas disease.

## Introduction

Chagas disease is an infection caused by the flagellate protozoan *Trypanosoma cruzi* (*T. cruzi*) and represents a significant public health burden in the Americas [1], [2]. The disease develops from an initial acute phase that progresses to an asymptomatic indeterminate period, with virtually undetectable parasitemia, as a result of non-sterile control of the parasite by strong humoral and cellular anti-*T. cruzi* responses. Several years after the initial infection, approximately 20 to 30% of all infected individuals develop a chronic inflammatory disease which primarily affects the heart [3]–[5].

The pathogenesis of Chagas disease is controversial and distinct hypothesis have been considered, including autoimmune manifestations and parasite-driven tissue damage [6]–[10]. In murine models of acute Chagas disease, alterations have been observed in lymphoid organs including the thymus, in which the parasite has been detected [11]. Infected animals reveal a severe atrophy of the organ, although not affecting the key intrathymic events responsible for negative selection of thymocytes during thymopoiesis [12]

Thymic atrophy is also seen in other infections and results from the inflammatory syndrome mediated by TNF-α during the acute phase of infection; this leads to activation of the hypothalamus-pituitary-adrenal (HPA) axis with the consequent release of corticosterone [13]. The rise in glucocorticoid levels during infection is related to the extensive apoptosis of immature CD4^+^CD8^+^ cells, which accounts for the atrophy of the thymus [13]–[15], together with the premature release of recent thymic emigrant cells, including CD4^+^CD8^+^ thymocytes [16]–[18]. Moreover, we found that increased percentages of circulating CD4^+^CD8^+^ T cells exhibiting an activated HLA-DR^+^ phenotype are associated with severe cardiac forms of human chronic Chagas disease [12]. However, it remains unknown if there is an involvement of CD4^−^CD8^−^ T lymphocytes in the pathogenesis of this infection. Extrathymic CD4^−^CD8^−^TCR^+^ cells that lack the expression of both CD4 and CD8 T cell co-receptors, are found in several immune disorders, representing key players in autoimmunity and inflammation [19]–[21].

The premature release of immature thymocytes in Chagas disease likely results from *T. cruzi*-induced alterations of the thymic microenvironment represented by an increased deposition of migration-related cell molecules, such the extracellular matrix (ECM) proteins, fibronectin and laminin, as well as the CXCL12 and CCL21 chemokines in the thymus [22]. Additionally, the infection induces an increase in the expression of the fibronectin/laminin integrin receptors VLA-4 and VLA-6 in thymocytes, resulting in an enhanced fibronectin/laminin-driven migration of thymocytes during the T cell maturation [23]. The cell-matrix-driven migration is settled by the interaction of thymocytes with thymic epithelial cells, as well as other cell types of the thymic microenvironment. This process is important for thymocyte migration within thymic compartments, along which the thymocytes become sensitive to regulation of exit by the chemo-attractant receptor CCR7 and the receptor for sphingosine-1-phosphate (S1PR1) [24], [25].

Sphingosine-1-phosphate, a breakdown product of sphingolipid metabolism, is present in all mammalian cells and serves as a second messenger in signal transduction pathways. Reversible systemic and local synthesis of S1P is mediated by sphingosine kinases (SPHK1/2) and S1P phosphatases (SGPP1/2), whereas irreversible degradation of S1P is carried out by a single enzyme, S1P lyase (SGPL) [26]. S1P is released into the extracellular milieu by a variety of cell types, making it one of the most abundant biologically active lysophospholipids in circulation [27]–[29].

Autocrine and paracrine interactions between S1P and a family of five different G protein-coupled receptors (S1PR1-5) have been implicated in a wide range of physiological activities, including immunity [30], [31]. It has been shown that the S1P receptor surface expression on lymphocytes is critical for their egress from thymus and lymph nodes. Increased surface expression of S1PR1 renders T cells responsive to S1P chemotactic signals and promotes their emigration from lymphoid tissues, whereas S1PR1 is downregulated during peripheral lymphocyte activation, and this fate is associated with cell retention in the lymphoid organs [24], [25], [32].

We investigated herein the role of the S1P pathway in *Trypanosoma cruzi*-induced thymus atrophy. Our findings indicate that the intrathymic levels of S1P kinase activity decrease in *T. cruzi* acutely infected mouse thymus. This is associated with upregulation of S1PR1 receptor expression and increased chemotactic responsiveness of CD4^−^CD8^−^ thymocytes to S1P. When acutely-infected animals were systemically treated with FTY720, a potent antagonist of the S1P receptor, the reduction of the thymic cellularity seen in *T. cruzi* infection was significantly abrogated and the intrathymic contents of CD4^−^CD8^−^ thymocyte population were recovered. Our data indicate that CD4^−^CD8^−^ thymocytes exhibit a pro-inflammatory like activation pattern, based on IL-17 and TNF-α expression gene profiles, and that the S1P signaling pathway exerts a critical role on the premature release of these cells during *T cruzi*-induced thymic atrophy. Finally, increased numbers of circulating CD4^−^CD8^−^ T cells were also found in chronic chagasic patients bearing severe cardiopathy.

## Materials and Methods

### Ethics statement

The study was approved by the Research Ethics Committee of National University of Rosario, (protocol UNR-CD N°2854/2008 and CS N°131/2010) and Fiocruz (protocol CEUA-LW8/10). Protocols for animal and human studies were approved by the Institutional Ethical Committees in accordance with international guidelines. All animal experimentation was performed in accordance with the terms of the Brazilian and Argentine guidelines for the animal welfare regulations. All subjects in the study were adult and provided a written informed consent.

### Study population

Healthy volunteers and chronic chagasic patients were recruited from Chagas Unit, Hospital Provincial del Centenario de Rosario, UNR, Argentina. Subjects using any medication thought to affect immune functions were excluded from the study. The diagnosis of *T. cruzi* infection was based on two standard serological tests including indirect immunofluorescence and haemaglutination assay. The ages of all chronic infected patients and healthy volunteers that participated in the study reanged from 30 to 70 years. Seropositive cases included eleven cardiac chronically-infected patients presenting dilated cardiomyopathy diagnosed based on a detailed clinical examination, as well as electrocardiography (ECG) and chest X-ray. Additionally, we included twelve chagasic patients without any cardiac alterations detected, being diagnosed as in the indeterminate or asymptomatic form of the disease. Ten sex and age matched-controls were also included.

### Animals, infection and drug treatment

Male BALB/c mice, aged 4–8 weeks, were obtained from the animal facilities of Oswaldo Cruz Foundation and Federal University of Rio de Janeiro or from the Faculty of Medical Sciences of National University of Rosario. Acute *T. cruzi* infection was performed by inoculating the animals intraperitoneally with 10^2^ blood-derived trypomastigote forms of the *Tulahuen* strain, after isolation from BALB/c mice. At different days post-infection, animals were killed and the organs to be studied were removed. Blood parasites were counted using Neubauer's chambers. For blocking the S1P-mediated egress of thymocytes, infected mice were treated with FTY720 (5.0 mg/kg), the functional antagonist for S1PR1 [33], every two days through was administrated *in vivo* through intraperitoneal injections from 6 dpi to 16 dpi. At day 17 dpi, animals were killed for further analysis.

### Lipid phosphorylation assay, extraction and analysis

To ascertain the activity of SPK activity we performed a lipid phosphorylation assay as previously described [34]. Briefly, we used 0.2 mg protein/ml from each sample and the assay medium (1 ml) contained 30 mM MES-Tris (pH 7.0), 1 mM [γ-^32^P]ATP (specific activity 2.2×10^8^ cpm/µmol), 1.1 mM MgCl_2_, 10 mM NaN_3_, 1 mM ouabain, and 0.5 mM EGTA. The reaction was started by the addition of [γ-^32^P]ATP to the tubes. One ml aliquots of the different samples at the different experimental conditions were used for total lipid extraction, which was performed as previously described [34]. Briefly, 5.0 ml of the solvent moisture (CHCl_3_∶MeOH∶HCl – 20∶10∶0.075 v/v) was added to the samples at conic glass tubes and vigorous mixed using a Pasteur pipete. After 10 min on ice, it was added 1.0 ml 0.6 M HCl followed by intense mix with the Pasteur pipette. The tubes were centrifuged at 600×*g* for 10 min, and the lower phase (containing the lipids) was carefully removed for another glass tube and washed twice with 1.0 ml of CHCl_3_: MeOH: 0.6 M HCl (3∶48∶47 v/v) followed by centrifugation at 600×*g* for 10 min. The upper phase was removed and the lower phase was dried under N_2_. Total lipid content was determined gravimetrically. Lipids were separated and first identified by TLC, using a solvent system consisting of chloroform∶acetone∶methanol∶acetic acid∶water (120∶45∶39∶36∶24, v/v). Then, the [γ-^32^P]ATP containing phosphorylated lipids were detected in the autoradiograms of the TLC plates. To quantify the amount of S1P formed, the corresponding spot was scrapped and placed in vials for liquid scintillation counting (Tri-Carb 2100; Packard).

### Immunohistochemistry

Immunofluorescence analysis was performed to assess the intrathymic expression of S1PR1. For this purpose, thymuses were removed from 14 dpi and uninfected mice, embedded in Tissue-Tek (Miles, Elkhart, IN) and frozen in liquid nitrogen. Five 4 µm-thick cryostat sections were settled on poly-L-lysine (Sigma, St. Louis, MO) covered glass slides, acetone fixed and blocked with 1% PBS-BSA. Samples were submitted to specific rabbit anti-mouse EDG1 antibody (Abcam, Cambridge, UK), 1∶50 for 2 hour at room temperature, washed and submitted to appropriate secondary antibody, 1∶500 goat anti-rabbit Alexa 488 (Molecular Probes, Eugene, USA) respectively. Negative control was obtained by omitting primary antibodies. Samples were analyzed by confocal microscopy using a LSM 510 Zeiss device (Germany) and the images obtained were subsequently analyzed using the Image J software (NIH, Bethesda, MD).

### Isolation of human peripheral blood cells (PBMC), antibodies and flow cytometry

Heparinized whole blood was collected and diluted 1/2 with PBS before separated by density centrifugation on Ficoll-Hypaque (Sigma) for 30 min at 2000 rpm. For human T cell phenotyping, individual samples contained 10^6^ living cells were pre-treated with human AB serum for 15 min and stained at 4°C for 30 min simultaneously with three colors using the following antibodies: APC-labeled anti-CD3, APCCy7-labeled anti-CD4, PECy7-labeled anti-CD8 antibodies (BD/PharMingen, CA). For FACS analysis, PBMC cells were then fixed and analyzed and CD3^+^ T lymphocytes were gated based on forward and side scatter parameters followed by CD3^+^ expression to avoid larger leukocytes such as macrophages and granulocytes.

For murine T cell phenotyping experiments, thymuses, subcutaneous lymph nodes and spleens from 6–10 week-old mice were dissected and mechanically disaggregated. Single cell suspensions were obtained using a 40 µm cell strainer. Red blood cells were removed by NH4Cl lysis. Cells were diluted in 1% BSA-PBS and incubated with anti-FcγR antibody (BD Biosciences, California, USA) to block the binding of conjugated antibodies to FcγR. Individual samples stained at 4°C for 30 min simultaneously with different combination of the following antibodies for FACS analysis: PerCP-labeled anti-CD3, PE-cy7-labeled anti-CD4, APC-labeled anti-CD8, FITC-labeled anti-CD11b, FITC-labeled anti-CD161 (NK T cell specific), PE-labeled anti-CD11c, PE-labeled anti-CD45, PE-labeled anti-TCRγδ. For the characterization of DN T cells TCR^+^, CD4 and CD8 staining was combined with the PE-labeled anti-TCR Vbeta reagent. All the antibodies were obtained from BD Biosciences. After staining, cells were then fixed and analyzed by FACS Canto II flow cytometer (Beckton Dickinson, CA). Analyses were done by duplicate, recording 25,000–50,000 events for each sample, then using the DIVA software (Becton Dickinson). Lymphocytes were gated based on forward and side scatter parameters, so as to avoid larger leukocytes such as macrophages and granulocytes.

### Isolation of thymic CD4^−^CD8^−^ cells and quantification of mouse mRNA transcripts

CD4^−^CD8^−^ T lymphocytes were isolated from three pooled thymuses obtained from infected (14 dpi) and uninfected (control) mice by FACS cell sorting using PE-cy7-labeled anti-CD4, APC-labeled anti-CD8. For quantification of mRNA transcripts, RNA was extracted from CD4^−^CD8^−^ enriched cells (5.10^5^ *per* group) using a commercially available kit (RNeasy Mini Kit, Qiagen, Venlo, NL). For total thymus analysis, RNA was isolated using guanidine thiocyanate kit (Invitrogen, California, USA) per the manufacturer's instructions. First strand cDNA synthesis was prepared with 0.5 µg total RNA, random hexamer primer, and SuperscriptIII reverse transcriptase (Invitrogen, California, USA). For qPCR we used approximately 60 ng of cDNA for each sample and SYBR Green Master Mix 2 (Applied Biosystems, California, USA). cDNA was amplified using specific murine primer sequences described in Table 1. All reactions were run on an ABI Prism 7700 sequence detection system (Applied Biosystems, Foster City, CA). After 40 cycles of amplification, expression of GAPDH (Gapdh), S1PK1 (Sphk1), S1PK2 (Sphk2), S1PP1 (Sgpp1), S1PP2 (Sgpp2), S1PL (Sgpl1), S1PR1 (S1pr1), S1PR3 (S1pr3), IL17 (Il17a) and TNFα (Tnf) was assessed by comparing the expression of each to the normalizer GAPDH using the Ct method as previously described (2^−ΔCt^) [35], subsequent to the following primer efficiency analysis. Each experiment was run in triplicate with different cDNA preps from the same mice. GenBank accession number for each of these genes can be seen in Table 1.

**Table 1:** General features of genes whose expression was evaluated by quantitative RT-PCR analysis*.

Symbol	GenBank	Description	Description
**Gadph**	NM_008084.2	Glyceraldehyde-3-phosphate dehydrogenase	CCGCCTGGAGAAACCTGCCAAGTAT (FW)
			TTGCTCAGTGTCCTTGCTGGGGT (RV)
**Sphk1**	NM_001172475.1	Shingosine kinase 1	GGAGGAGGCAGAGATAACCTT (FW)
			GACCCAACTCCCTGCACACA (RV)
**Sphk2**	NM_001172561.1	Sphingosine kinase 2	GCCCGAGATGGTCTAGTCT (FW)
			GTGGGTAGGTGTAGATGCAGA (RV)
**Sgpp1**	NM_030750.3	Sphingosine-1-phosphate phosphatase 1	GGGTGCTGGTCATGTACCTG (FW)
			CCCGTAGATAAGAGGATAGTGCC (RV)
**Sgpp2**	NM_001004173.2	Sphingosine-1-phosphate phosphatase 2	GTTCTCTACGCTGGTGTGTCT (FW)
			GCAGGGTAGGTGAGAGCAAT (RV)
**Sgpl1**	NM_009163.3	Sphingosine phosphate lyase 1	CCTGTGTACTCTGCTGATAGTCT (FW)
			CTGTTGTTCGATVVTACGTCCA (RV)
**S1pr1**	NM_007901.5	Sphingosine-1-phosphate receptor 1	GTGGGCTGCAAGGTGAAGACCTGT (FW)
			TTTCTGGGGGTGGGAGGAATTGTC (RV)
**S1pr3**	NM_010101.4	Sphingosine-1-phosphate receptor 2	GCCCATCCTCTTCAAGGCTCAGT (FW)
			GTGGGGCAGGTCTTCCTTGACCTT (RV)
**Il17a**	NM_010552.3	Interleukin 17a	TCATCCCTCAAAGCTCAGCG (FW)
			TTCATTGCGGTGGAGAGTCC (RV)
**Tnf**	NM_013693.2	Tumor necrosis factor	TGTCTACTGAACTTCGGGGT (FW)
			TCCACTTGGTGGTTTGCTAC (RV)

* Symbols, GenBank accession numbers and descriptions of the genes mentioned in the text, as well as the corresponding nucleotide sequences of primers used for the analysis of transcripts.

### Evaluation of recent thymic emigrants

Intrathymic injection of fluoresceinisothiocyanate (FITC) for direct evaluation of recent thymic emigrants (RTEs) in the mouse model were performed as originally described by Scollay et al [36] with minor modifications. Briefly, animals were anesthetized (ketamine at 100 mg/kg and xylaxine at 2 mg/kg) and then the chest was opened to expose the thymus. A Hamilton syringe was used to inject 10 µL/lobe of 1 mg/ml FITC solution, prepared from a saturated stock diluted in phosphate-buffered saline. Control group was injected with saline alone. After intrathymic injection, the skin incision was closed and mice were allowed to recover under a heat source. To evaluate RTEs, animals were sacrificed 24 hours post-injection, and thymus, spleen as well as axillary and inguinal subcutaneous lymph nodes were removed. Lymphoid cells from each organ were suspended in PBS solution containing 5% fetal calf serum; being then labeled with PE-labeled anti-CD3, APC or PECy7-labeled anti-CD4, PECy7or APCCy7-labeled anti-CD8 antibodies (BD/PharMingen) for monitoring FITC^+^ T cells by flow cytometry. To determined DN stages we also stained cells with PECy5-labeled anti-CD44 and APC-labeled anti-CD25 antibodies (BD/PharMingen). Only animals with more than 50% of FITC^+^ thymocytes were used for subsequent analysis. Peripheral cells were considered RTEs when FITC^+^ cells could be distinguished from FITC^−^ cells, comparing with animals that only received intrathymic injection of saline. Data were evaluated as absolute numbers or percentages of FITC^+^ cells present in the perypheral lymphoid organs.

### Chemotaxis assay

The response of thymocytes towards sphingosine-1-phosphate was studied using Transwell inserts with a 5 µm pore size (Corning, Cambridge, MA). Briefly, unspecific binding sites of the inserts were blocked with fatty-acid free PBS/BSA 0.1% (Sigma –Aldrich, St. Louis, MO) during 15 min at 37°C. Unbound BSA was then removed before adding thymocytes. The migratory response of thymocytes obtained from 14 dpi infected mice was analyzed under S1P concentrations of 0.01, 0.1, 1, 10 and 100 nM and BSA was used as control. Further, CD4^−^CD8^−^ and CD4^+^CD8^+^ T cells were isolated at 14 days post-infection or from uninfected counterparts by cell sorting and their migration was assessed under a fixe 0.01 nM concentration of S1P. Cell suspensions (100 µl at 5×10^5^/ml) in RPMI 1640 medium plus 0.4 mg/ml fatty acid-free BSA (Sigma) were added to the upper chambers. Each insert was placed in a well containing 600 µl of a solution of sphingosine-1-phosphate (Avanti), 0.1 nM, prepared in medium containing fatty acid-free BSA. Wells containing medium in the presence of fatty acid-free BSA without sphingosine-1-phosphate were used as controls. After 3 h at 37°C, cells in the wells were harvested, counted, and analyzed by flow cytometry. The percentage of each thymocyte subset was assessed from absolute cell counting to assess the total numbers of each CD4^−^CD8^−^ and CD4^+^CD8^+^ subset. The specificity of CD4^−^CD8^−^ T cell migration towards S1P, was confirmed by the previous incubation of these cells with FTY720, 10^2^ nM, during 30 min at 37°C. Both S1P and FTY720 working concentrations were selected after the obtainment of a dose-response curve.

### *In vitro* activation of CD4^−^CD8^−^ T cells

To access the cytokine-producing *in vitro*, CD4^−^CD8^−^ enriched lymphocyte suspensions (5×10^5^ cells), obtained from thymuses of both uninfected and 14 dpi mice, were cultured in lymphocyte culture medium supplemented with 10 ng/mL phorbol 12-myristate 13-acetate (PMA) 500 ng/mL ionomycin and 5 µg/mL of brefeldin A (all from Sigma-Aldrich) for 3 hours, at 37°C. The cells were then recovered and ressuspended in a lysis buffer, prior to RNA isolation (RNeasy Mini Kit, Qiagen, Venlo, NL).

### Statistical analysis

Statistical analyses were performed with GraphPad Prism 4 software, using one-way ANOVA test. In humans, we used non-parametric tests. Non-parametric Kruskall-Wallis test (k groups >2) followed by the U Mann-Whitney test (k groups = 2). Results were expressed as mean ± standard error (S.E.). Differences between control versus treated group were considered statistically significant when *P*<0.05.

## Results

### *Trypanosoma cruzi* infection promotes abnormal release of immature thymocytes

**Figure 1:**
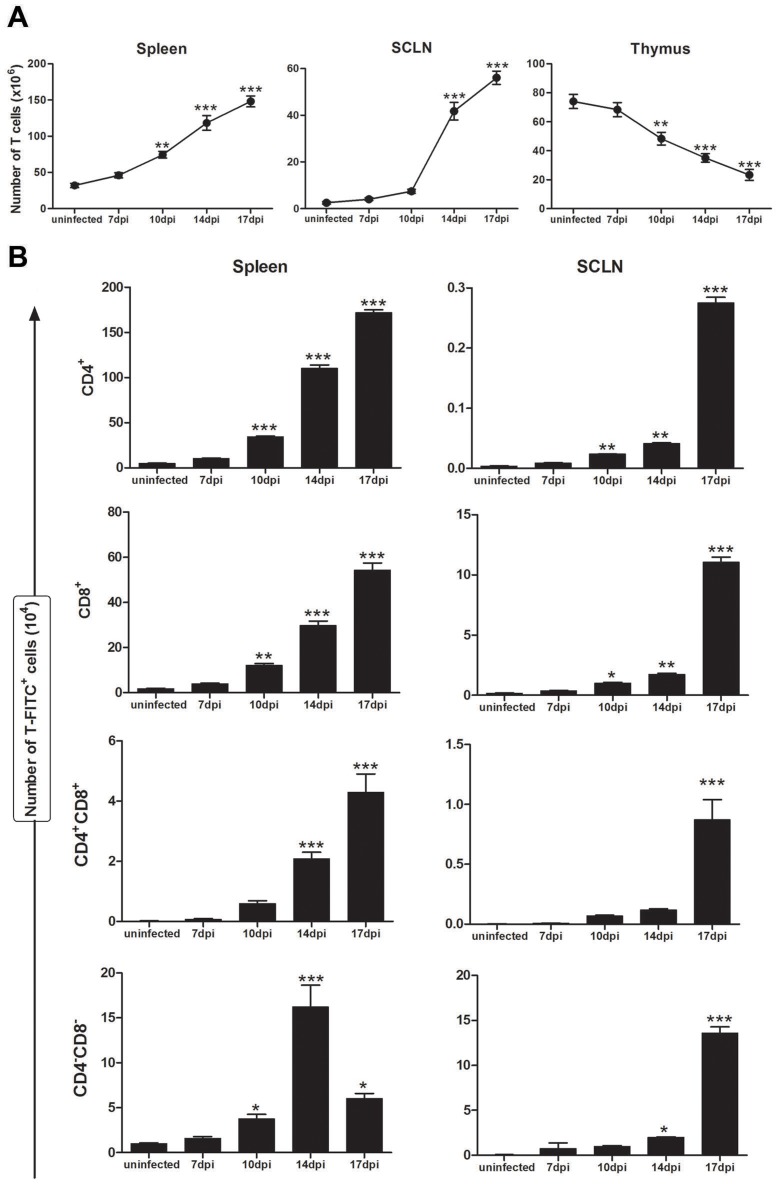
*Trypanosoma cruzi* acute infection promotes the premature release of CD4^−^CD8^−^ thymocyte cells to the periphery. BALB/c mice were intrathymically injected with FITC solution or PBS only as a control. After 24 h, lymphoid tissues were harvested and cells stained with anti-CD4 and anti-CD8, for the identification of the specific subsets of recent thymic emigrants (RTEs) by flow cytometry. (**A**) Graphs represent the absolute numbers of total T cells in the thymus, spleen and subcutaneous lymph nodes (SCLN) during *T. cruzi* acute infection and uninfected mice. (**B**) Histograms represent the total numbers of RTEs (FITC^+^ cells) bearing the CD4^+^, CD8^+^, CD4^+^CD8^+^ and CD4^−^CD8^−^ T cell phenotype, obtained from the spleen (**right panel**) and SCLN (**left panel**), as indicated. Data are shown as means ± SE (n = 6 mice per group). Differences between uninfected and infected mice are significant * (*p*<0.05), ** (*p*<0.01), *** (*p*<0.001).

In the mouse model of acute *T. cruzi* infection, BALB/c mice were injected intraperitoneally with 10^2^ *Tulahuén* trypomastigote parasites. This induced severe thymic atrophy that is evident 10 days post-infection, is mainly due to the CD4^+^CD8^+^ double positive loss (Figure S1). As expected, we also found a marked increase in the number of total splenic and subcutaneous lymph node T cells in such *T. cruzi*-infected mice (Fig. 1A). Conversely, in the thymus itself there was a significant reduction in the total thymocyte numbers from day 7 post-infection to the late stage of acute infection (Fig. 1A and Figure S2).

To confirm that the atrophy in *T. cruzi*-infected mice was also related to the premature release of thymocytes we labeled thymocytes by intrathymic injection of fluorescein isothiocyanate (FITC) (Figure S2). On the day after FITC injection, the numbers of peripheral FITC^+^ cells expressing the CD3 T cell marker were determined in the lymphoid organs. We found evidence of an abnormal escape of both immature CD4^−^CD8^−^CD3^+^FITC^+^ and CD4^+^CD8^+^CD3^+^FITC^+^ to the periphery during infection, as indicated by the numbers of RTEs detected in the spleen and lymph nodes at 10 dpi (Fig. 1B). There was also as increase in the absolute numbers of conventional CD4^+^ and CD8^+^ single-positive T cells in the peripheral lymphoid tissues analyzed (Fig. 1B). Strikingly, higher proportion of FITC^+^ bearing DN cells CD4^−^CD8^−^CD44^−^CD25^+^ (DN3) and CD4^−^CD8^−^CD44^−^CD25^−^ (DN4) cells were observed in the spleen of infected animals (Figure S3).

### Modulation of the thymic sphingosine pathway in *T. cruzi* acute infection

**Figure 2:**
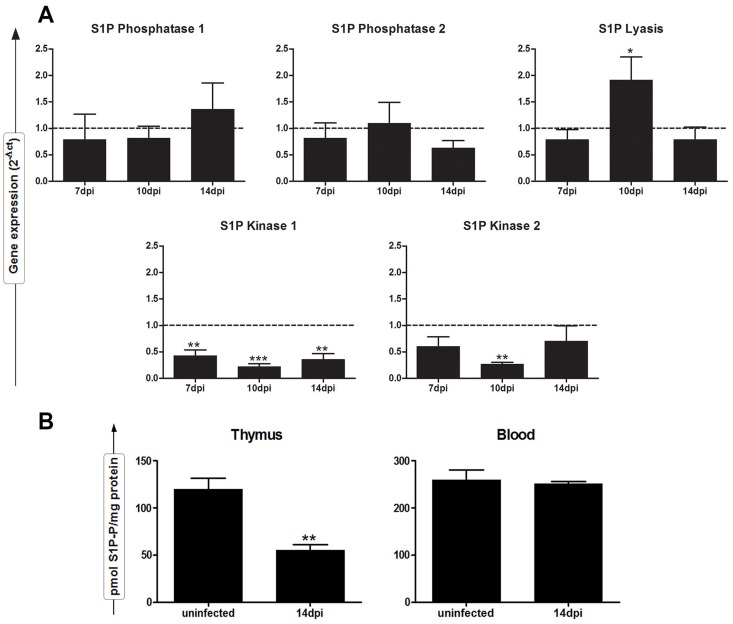
Down-modulation of intrathymic levels of S1P kinase activity in *T. cruzi* acutely-infected mice is associated with catabolic gene expression of the S1P pathway. Thymuses were collected from normal or *T. cruzi*-infected BALB/c on days 7, 10 and 14 post-infection (dpi) for measurements of the expression of S1P metabolic genes by real-time PCR and the levels of S1P levels by TLC. (**A**) mRNA levels of murine S1P kinases (SPHK1/2), S1P phosphatases (SGPP1/2) and the S1P lyase (SGPL1) from normal and *T. cruzi*-infected thymuses. Quantitative PCR results were normalized to values obtained from uninfected thymuses. Gene expression was assessed by comparing the expression of each to the normalizer GAPDH using the Ct method and is represented as mean ± SE of three independent experiments (n = 3 mice per group). (**B**) S1P kinase activity in thymuses and sera from normal or infected mice were measured by TLC, as described in Materials and Methods. C_17_-S1P was used as the internal standard. Graphs show the average values from two independent experiments (n = 6 mice *per* group), with error bars representing the standard error. Differences between uninfected and infected mice are significant * (*p*<0.05), ** (*p*<0.01), *** (*p*<0.001).

We next investigated whether the early release of thymocytes in *T. cruzi* acute infection could be favored by the modulation of the sphingosine-1-phosphate (S1P) pathway, which is critical to license mature thymocytes for trafficking competence and emigration from the thymus. A dynamic balance between S1P and its precursors, ceramide and sphingosine, is an important factor that determines thymocyte fate [31]. Sphingosine-1-phosphate is generated by the phosphorylation of sphingosine, catalyzed by two sphingosine kinases, SPHK1 and SPHK2. It is inactivated through reversible dephosphorylation by two phosphatases, SGPP1 and SGPP2, and through definitive degradation by the lyase, SGPL1. To address this issue, gene expression profiles of the enzymes involved in the S1P pathway, were compared between normal versus *T cruzi*-infected thymus during acute phase at days 7, 10 and 14 post-infection by using quantitative real-time PCR (qPCR). As shown in Figure 2A, SPHK1/2 expression was significantly reduced in the thymuses from infected animals as compared to non-infected controls. In contrast, there was an up-regulation of the SGPL1 at 10 dpi, whereas the expression of SGPP1/2 remained unaltered in *T cruzi*-infected thymuses compared to the normal expression seen in the control-uninfected group. These findings suggest that S1P catabolism may be activated upon *T cruzi*-infection. In fact, TLC analysis of lipids present in both infected and normal thymuses indicated that in acutely *T cruzi*-infected thymus, there is a significant decrease in the S1P kinase activity when compared to the physiological condition (Fig. 2B).

Despite the differences in the thymus, serum S1P levels were comparable in both infected and normal mice (Fig. 2B). These results indicate an increased gradient in the S1P levels from thymus to the blood during the *T. cruzi*-induced thymic atrophy that may favor the exit of thymocytes in the infection.

### The S1PR1 and S1PR3 receptors are up-regulated in the double-negative thymocytes from *T cruzi*-infected thymuses and following chemotaxis to lower doses of S1P

**Figure 3:**
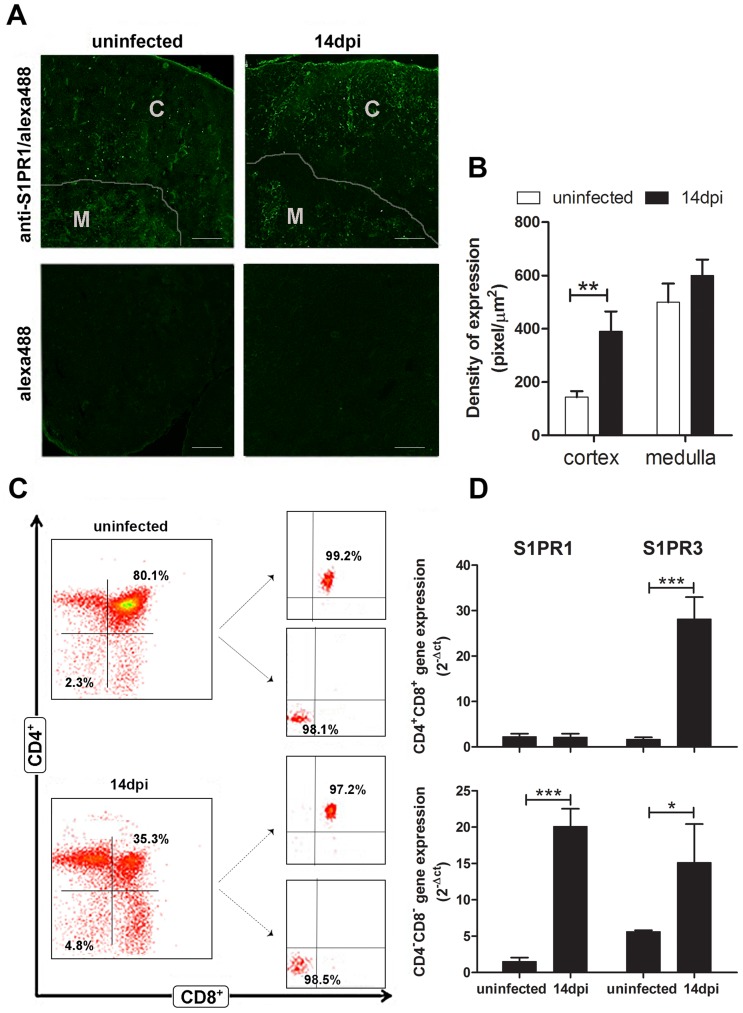
*T. cruzi* acute infection increases expression of S1P receptors in CD4^−^CD8^−^ thymocytes and thymic cortical compartment. (**A**) Thymuses were stained with anti-S1PR1 monoclonal antibody and analyzed by immunofluorescence. Representative confocal microscopy fields of thymuses stained with anti-S1PR1 monoclonal antibody and goat-anti rabbit alexa-488 secondary antibody are shown in (upper left) normal thymus and (upper right) atrophic thymus at day 14 post-infection. The respective staining controls without the primary antibodies are shown in (lower left) and (lower right). Scale bar = 100 µm. (**B**) Graphs correspond to relative quantitation analysis of S1PR1 deposition from 5 microscopic fields of thymuses from control (n = 3) or infected animals (n = 3). It is clear the increase in S1PR1 within the cortex of thymic lobules from infected animals. (**C**) CD4^−^CD8^−^ and CD4^+^CD8^+^ T cells from normal and acutely-infected mice (14 dpi) were purified from thymus, by cell sorting using flow cytometry to assess the transcriptional levels of S1P receptors. Plots show the high purity of isolated CD4^−^CD8^−^ and CD4^+^CD8^+^ T cells (>95%). (**D**) Total mRNA from purified CD4^−^CD8^−^ and CD4^+^CD8^+^ T cells were processed for RT-PCR. The results represent the amount of transcripts, depicted in relation to the housekeeping gene GAPDH as 2^−ΔCt^. There is a significant increase in the contents of both S1PR1and S1PR3 transcripts when comparing control *versus* infected CD4^−^CD8^−^ T cells, whereas only S1PR3 transcripts increased in the CD4^+^CD8^+^ T subset relative to the controls. Data are means ± SE of two independent experiments using six mice *per* group. Differences between control and infected mice are significant * (*p*<0.05), ** (*p*<0.01) and *** (*p*<0.001).

We further analyzed whether the modulation of the S1P pathway also comprised the thymic expression S1PR1 and S1PR3, the major S1P receptors involved in S1P-induced T cell migration [37], [38]. As demonstrated in Figure 3A and B, S1PR1 was higher in the cortical thymic compartments of infected animals (14 dpi) than in the corresponding regions of non-infected control mice. Yet, we did not detect any alteration in the S1PR3 protein expression levels (data not shown).

We next assessed the expression of the S1P receptor in developing thymocytes. To this end, both CD4^−^CD8^−^ and CD4^+^CD8^+^ cells were purified by FACS (Fig. 3C) and S1PR1 and S1PR3 gene expression levels were determined by real-time RT–PCR analysis (Fig. 3D). We found a substantial up-regulation of the S1PR3 mRNA in immature CD4^−^CD8^−^ as well as CD4^+^CD8^+^ thymocytes from the infected mice, as compared with their uninfected counterparts (Fig. 3D). Furthermore, there was an increase in S1PR1 expression in CD4^−^CD8^−^, but not in CD4^+^CD8^+^ T cells (Fig. 3D), pointing to different mechanisms driving the exit of these two immature thymocyte subsets. These findings suggest that an S1P-mediated pathway may regulate the premature exit of immature thymocyte cells from the thymus, particularly the CD4^−^CD8^−^ thymocytes (Fig. 3D).

**Figure 4:**
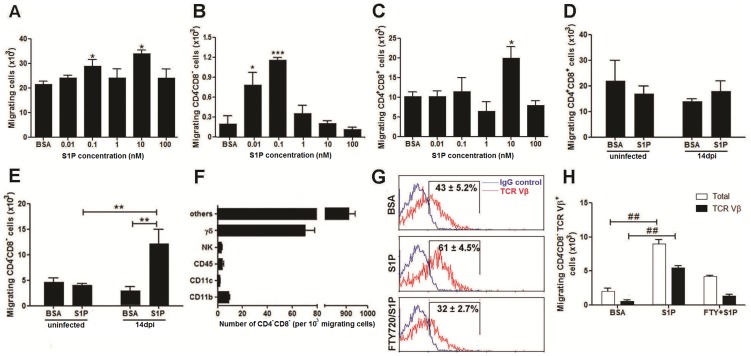
S1P promotes migration of immature thymocytes from *T. cruzi-*infected mice. Migratory responses of thymocytes isolated from infected and normal mice were assessed in transwell chambers as described in Materials and Methods. S1P was added to the lower chamber at the indicated concentrations. (**A**) Total thymocytes obtained from infected mice were assayed at 0.01, 0.1, 1, 10 and 100 nM S1P. Our data show two peaks of migration, in 0.1 and 10 nM S1P. Phenotype characterization of migrating cells by FACS analysis showed that the higher migratory response of CD4^−^CD8^−^ thymocyte subset occurs towards 0.1 nM S1P (panel **B**), whereas CD4^+^CD8^+^ thymocyte subset migrates mainly through 10 nM stimuli of the agonist (**C**). Data show mean ± SE in four independent experiments using three pooled thymuses. Differences between S1P and BSA groups are significant # (*p*<0.05) and ### (*p*<0.001). The migratory responses of FACS isolated CD4^+^CD8^+^ (**D**) and CD4^−^CD8^−^ (**E**) T cells from uninfected and infected mice were assessed in transwell chambers towards 0.1 nM S1P stimulus. Our data show an increased S1P-driven migration of CD4^−^CD8^−^ thymocytes obtained from infected mice present as compared to non-infected controls. Data show mean ± SE in three independent experiments using five pooled thymuses. Differences between uninfected and infected groups are significant ** (*p*<0.01). Differences between S1P and BSA groups are significant ## (*p*<0.01). (**F**) Migrating CD4^−^CD8^−^ cells were characterized by flow cytometry analysis according to the expression of the following phenotypic markers: CD11b (macrophages), CD11c (dendritic cells), CD45 (B cells), CD161 (NK T cells) and TCRδ; Intrathymic CD4^−^CD8^−^ T cell progenitors (others) were characterized by the absence in the expression of these markers. Data show means ± SE per 10^3^ cells characterized in two independent experiments. (**G**) The determination of the S1PR1-dependent migration of CD4^−^CD8^−^ T cells isolated from infected mice was assessed upon treatment of the cells with the functional antagonist of S1P receptors FTY720 (10^2^ nM) during 30 min at 37°C previously to migration in transwell chambers towards the 0.1 nM S1P stimulus. A representative FACS profile is shown from one of three independent experiments. The ratio of CD4^−^CD8^−^TCR Vβ^+^ cells was obtained and the background staining eliminated using the isotype control antibody. (**H**) We show an increase in the absolute numbers of total and TCRVβ^+^CD4^−^CD8^−^ T cell migration toward the S1P stimulus. This is prevented when the cells were previously incubated with FTY720. Data show mean ± SE in two independent experiments using five pooled thymuses. Differences between S1P and BSA groups are significant ## (*p*<0.01).

Next we assessed the sensitivity of thymocytes to S1P-mediated chemotaxis during *T. cruzi*-induced thymus atrophy. Using the *ex-vivo* transwell migration assay, we first analyzed the chemotactic response of total thymocytes at day 14 after *T. cruzi* infection from 0.01 to 100 nM S1P. The results show significantly higher sensitivity of the migratory response of thymocytes from the infected animals, exhibiting a double-peak at 0.1 and 10 nM doses of S1P (Fig. 4A).

Considering the increased expression of the S1P receptors in immature thymocytes upon *T. cruzi* infection, we tested the importance of S1P-mediated chemotaxis in these cells. Phenotype characterization of total migrating cells obtained by FACS analysis showed the migratory response of CD4^−^CD8^−^ T cells peaks at lower concentration of the S1P stimuli (0.1 nM) than in the CD4^+^CD8^+^ T cells (10 nM), as represented respectively in Figures 4B and C. The higher sensitivity of the CD4^−^CD8^−^ cells to S1P-mediated chemotaxis is likely due to the augmented expression of both receptor types S1PR1 and S1PR3 in comparison to CD4^+^CD8^+^ cells, which increased only the S1PR3 receptor during *T. cruzi* infection (Fig. 3D).

The lower dose of S1P may have an effect during the *T. cruzi* infection as we show a steeper decrease in the concentration of this chemotatic agonist in the thymus following infection (Fig. 2B). In this regard, our data suggest that the disturbance of the physiological thymic levels of S1P have a target effect on the CD4^−^CD8^−^ thymocytes during *T. cruzi* infection. To address this issue, CD4^−^CD8^−^ and CD4^+^CD8^+^ thymocytes were purified by cell sorting from both normal and chagasic thymuses at day 14 post-infection and the role of S1P receptors in mediating cell chemotaxis to S1P was compared on a per cell basis by using an *in vitro* transwell migration assay. This effectis not seen in the normal thymus, since we found that at the lower S1P agonist concentration (0.1 nM) neither CD4^+^CD8^+^ nor CD4^−^CD8^−^ cells isolated by cell sorting from non-infected control thymuses responded to the S1P *stimuli* as the basal migration values obtained when BSA was applied were similar to those seen in the presence of S1P (Fig. 4D and E).

We then aimed to characterize the CD4^−^CD8^−^ thymocyte subset from *T. cruzi*-infected animals responding to S1P. By using multicolor cytofluorometric analysis after immunostaining with major cell surface markers that are known to characterize thymocyte subsets, our results indicated that the majority (>90%) of the migrating CD4^−^CD8^−^ thymocytes purified from *T. cruzi*-infected animals responding to the S1P-driven chemotaxis were identified as potential CD4^−^CD8^−^ T cells (Figs. 4F and 4G). Actually, roughly 70% of migrating CD4^−^CD8^−^ thymocytes exhibited membrane expression of the TCR Vβ protein. We further showed that the migration of these CD4^−^CD8^−^TCRVβ^+^ T cells towards S1P agonist was blocked using the FTY720 specific inhibitor [33] of the cognate S1P receptors (Figs. 4G and 4H).

### S1P is critical for determining CD4^−^CD8^−^ thymocyte egress during *T. cruzi* infection

**Figure 5:**
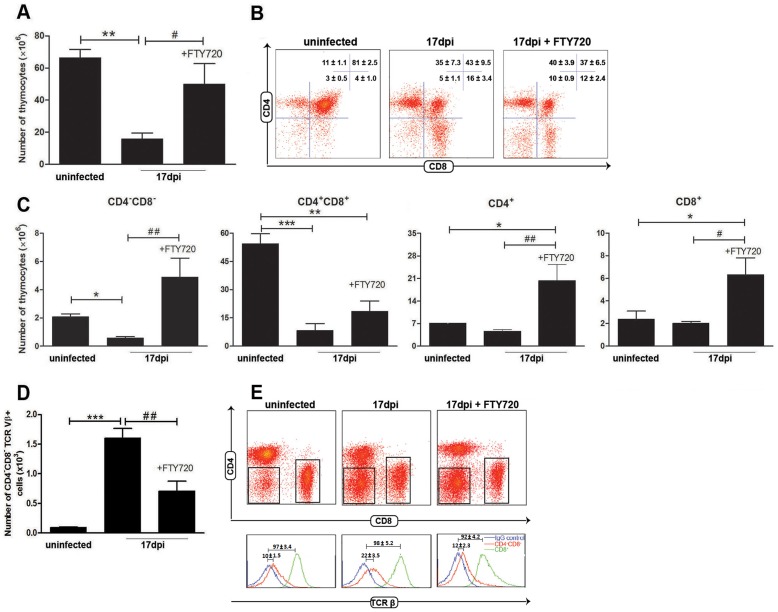
Treatment with FTY720 promotes thymic sequestration of CD4^−^CD8^−^ T cells with reduction of the corresponding peripheral pool during *T. cruzi* infection. Infected mice had been pretreated with FTY720 or saline every 2 days, starting at day 6 post-infection with *T. cruzi* parasites. At day 17 post-infection, thymocytes were harvested and (**A**) absolute cell numbers were determined by direct counting. (**B**) The cytofluorometric profiles of thymocytes were obtained after staining with anti-CD4 and anti-CD8 mabs. A representative FACS profile is shown and the ratio of each thymocyte population given as means ± SE. (**C**) The absolute numbers of CD4^−^CD8^−^, CD4^+^CD8^+^, CD4^+^ and CD8^+^ thymocytes were determined. CD4^−^CD8^−^ T cells are increased in FTY720-treated mice by 500%, when compared to infected untreated mice. (**D**) The accumulation of CD4^−^CD8^−^TCRVβ^+^ T cells in the SCLN during infection was significantly reduced in infected mice treated with FTY720. Data show a means ± SE in two independent experiments using five pooled thymuses. (**E**) Representative FACS profile of cells from SCLN after staining with anti-CD4 and anti-CD8 mabs (**upper panel**). Histograms represent TCRVβ expression in both immature CD4^−^CD8^−^ T cells (TCR negative/low) and CD8^+^ mature thymocytes (TCR high). The background staining was defined using the isotype control antibody. Differences between uninfected and infected groups are significant * (*p*<0.05), ** (*p*<0.01) and *** (*p*<0.001). Differences between FTY720-treated and untreated infected groups are significant # (*p*<0.05) and ## (*p*<0.01).

The data showed above suggested that S1P-triggered chemotaxis contribute to CD4^−^CD8^−^ T cell egress from the thymus during the acute phase of *T cruzi* infection. We then determined whether the disturbance of the thymic S1P pathway seen in *T. cruzi* infection could play any role in the export of CD4^−^CD8^−^ thymocytes during atrophy of the thymus. To test this hypothesis we injected mice with the FTY720 compound, the S1P receptor inhibitor [33]. As we have previously demonstrated, *T. cruzi* infection leads to atrophy of the thymus, which is evident after day 10 post-infection [12], [39], with a significant decrease in cell numbers when compared with uninfected mice (Fig. 5A). In contrast, *T. cruzi* infected mice treated with FTY720 in doses administered intraperitoneally every 2 days from day 6 up top day 16 post-infection, partially reverted the thymic cellularity (Fig. 5A). This phenomenon occurred without any alterations in the frequency of CD4^+^CD8^+^ compartment (Fig. 5B and C), the major target cells in the *T. cruzi* induced thymic atrophy. By contrast, we observed a significant increase in the amounts of CD4^−^CD8^−^ cells as well as CD4^+^ and CD8^+^ single-positive thymocytes (Fig. 5C).

In parallel to the thymic atrophy and reduction of immature thymocytes, during *T. cruzi* infection there is an increase in the numbers of CD4^−^CD8^−^Vβ^+^ T cells in peripheral lymphoid organs (Fig. 5D). In contrast, *T. cruzi* infected mice treated with FTY720, significantly reverted the increase in the amounts of CD4^−^CD8^−^ T cells seen in peripheral lymphoid organs of infected mice (Fig. 5D), indicating cell sequestration in the thymus, secondary to the blockage of the S1P pathway (Fig. 5C). The mean fluorescence intensity of the TCR Vβ was clearly low in CD4^−^CD8^−^ T cells, when compared to CD8^+^ mature cells expressing high TCR membrane levels (Fig. 5E).

### Peripheral CD4^−^CD8^−^ T cells in *Trypanosoma cruzi* infection express mRNA levels for TNF-α and IL-17

**Figure 6:**
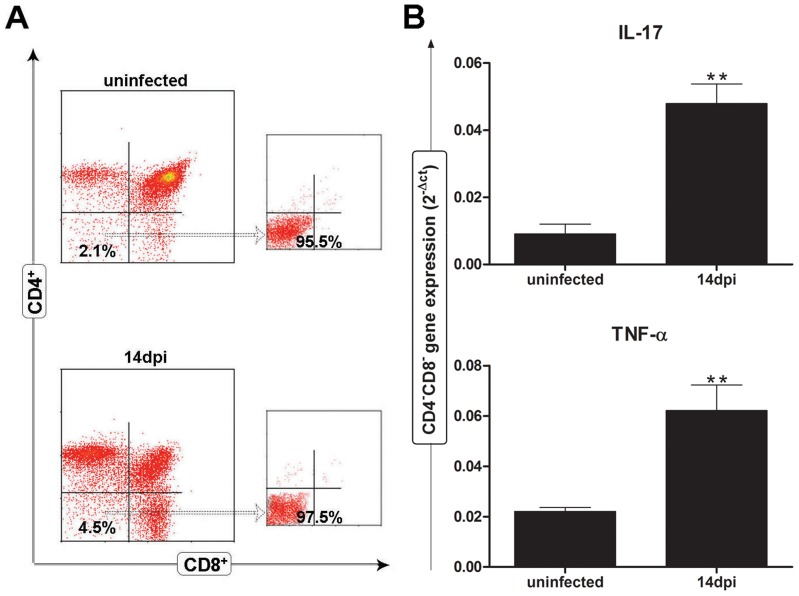
mRNA levels for TNF-α and IL-17a of CD4^−^CD8^−^CD3^+^ T cell subset in *T. cruzi* infected mice. (**A**) CD4^−^CD8^−^ T cells purified by cell sorting using flow cytometry from 3 pooled thymuses from control and *T. cruzi* infected mice at day 14 post-infection to a purity higher than 95% to access (**B**) the transcriptional levels of the pro-inflammatory cytokine TNF-α and IL-17a genes after *in vitro* stimulation with phorbol 12-myristate 13-acetate (PMA) and ionomycin in the presence of brefeldin A, during 3 hours at 37°C. The cytokine transcripts were assessed by qPCR and normalized to GAPDH. Data show the means ± SE in two independent experiments. Differences between uninfected and infected groups are significant ** (*p*<0.01).

Since our findings indicated that CD4^−^CD8^−^ thymocytes are abnormally released from the thymus in *T. cruzi* infection, we then attempted to demonstrate whether these cells could have an activation profile, as ascertained by the regulation of cytokine genes upon stimulation. To address this question, thymic CD4^−^CD8^−^ cells were purified by FACS cell sorting from normal or infected animals 14 days after infection (Fig. 6A), and total RNA was isolated after polyclonal activation with phorbol myristate acetate (PMA) and ionomycin. Quantitative PCR analysis revealed a significant gene expression induction of the cytokines IL-17 and TNFα in CD4^−^CD8^−^ T cells from *T. cruzi* infected mice, as compared to double-negative thymocytes purified from uninfected control mice (Fig. 6B).

### Abnormal high contents of CD4^−^CD8^−^ T cells in peripheral blood from chronic chagasic patients

In the light of the evidence described above, in the last set of experiments we searched for the presence of peripheral CD4^−^CD8^−^ T cells in human Chagas disease, particularly in patients bearing severe cardiomyopathy. For this purpose, we examined the frequencies of peripheral blood CD4^−^CD8^−^ T cells in cross-sectional studies of chronic chagasic patients at the indeterminate or cardiac clinical forms of Chagas disease, compared to age/sex-matched healthy individuals.

**Figure 7:**
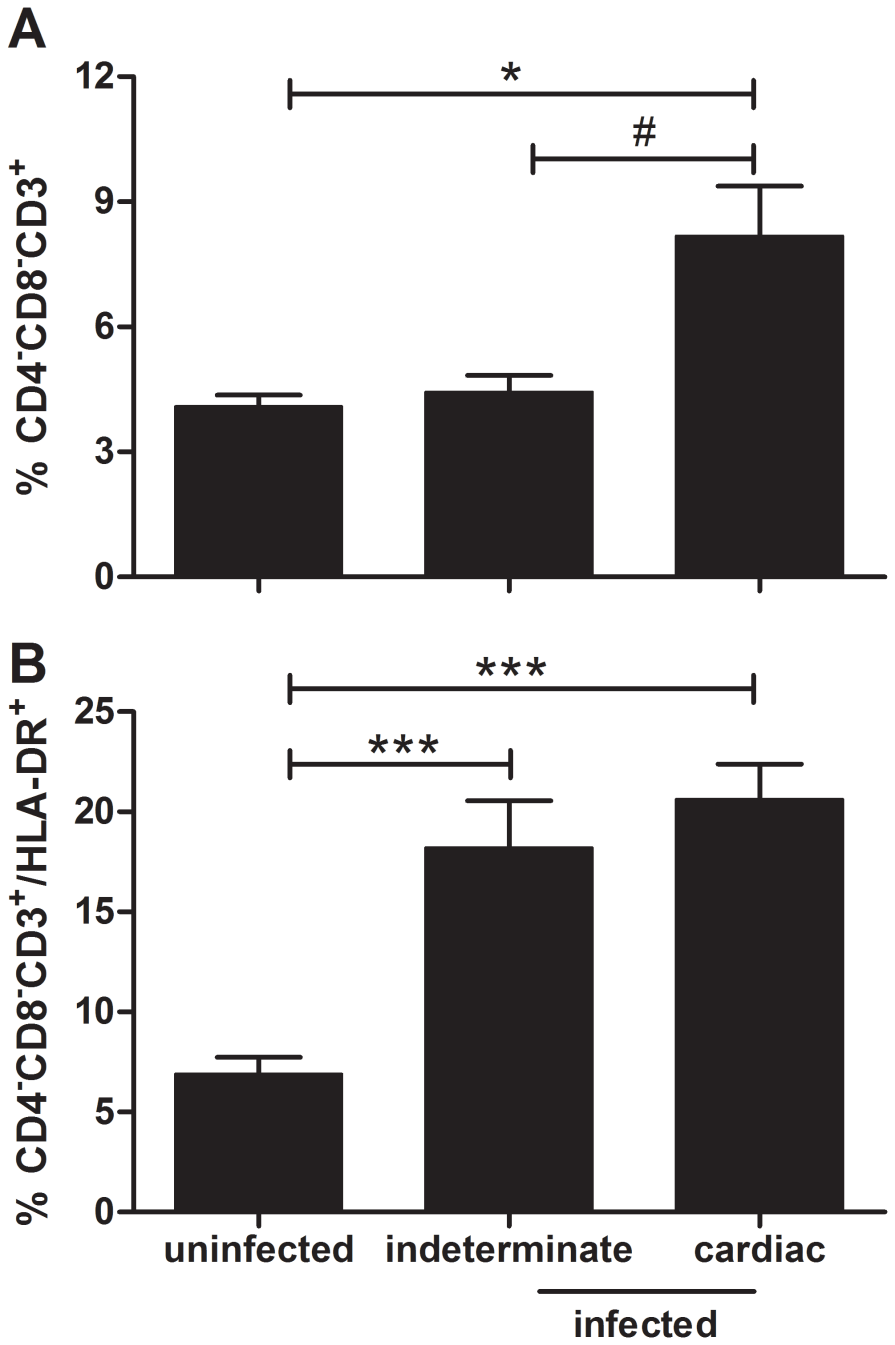
Increased numbers of CD4^−^CD8^−^ T lymphocytes in peripheral blood from chronic chagasic patients. Peripheral whole blood from non-infected individuals, patients at the indeterminate and cardiac forms of Chagas disease were analysed by four-color flow cytometry for expression of CD3, CD4, CD8 and HLA-DR markers. (**A**) The frequency of circulating human CD4^−^CD8^−^ T cells was measured in peripheral blood from non-infected individuals (n = 10), patients at the indeterminate (n = 12) and cardiac forms of Chagas disease (n = 11). Cells were analyzed by three-color flow cytometry for expression of CD3, CD4 and CD8. The percentages of CD4^−^CD8^−^CD3^+^ T lymphocytes are indicated for each histogram. (**B**) The activation profile of CD4^−^CD8^−^CD3^+^ T are shown by assessing the expression of HLA-DR. The significant differences between the groups were considered with # (*p*<0.05), * (*p*<0.05) and *** (*p*<0.001).

We found a significant increase in the relative numbers CD4^−^CD8^−^ T cells in cardiac chagasic patients, as compared to noninfected individuals. Such an increase was not seen in patients at the indeterminate form of the chronic disease (Fig. 7A). These results indicate the positive correlation between the frequencies of circulating CD4^−^CD8^−^ T cells and cardiomyopathy in chronic human Chagas disease. Moreover, we showed there is a statistically significant increase in the activation profile of extrathymic circulating CD4^−^CD8^−^ T cells in chagasic patients as compared to normal individuals by assessing the expression of activation marker HLA-DR from peripheral blood cells (Fig. 7B).

## Discussion

This study documents the effects of a well-characterized selective agonist sphingosine 1-phosphate upon thymic atrophy and immature T cell release during *T. cruzi* acute infection. In the thymus, endothelial cells as well as thymic epithelial cells are the major sources of extracellular S1P [40]–[42]. Sphingosine 1-phosphate levels are known to be very high (0.1–1 µM) in the blood, relative to other tissues, and to directly stimulate the migration of mature thymocytes out of the thymus via sphingosine-1-phosphate-specific chemotactic receptors [24], [32]. Although the concentration of plasma S1P is very high, it does not detectably enter the thymic compartments due to a S1P transporter in vascular endothelial cells that maintains high levels of S1P in the plasma in parallel with low levels within the thymic parenchyma [29]. SIP is also kept in low concentration in thymic tissue by decreased sphingosine kinase and augmented S1P lyase [26], [32]. Herein we demonstrated an imbalance of the S1P pathway in the thymus during *Trypanosoma cruzi* acute infection in mice. Our findings indicate a concomitant reduction in the transcriptional levels of the S1P kinases 1/2 together with an upregulation of the lyase gene. The transcriptional levels of the metabolic genes involved in the S1P pathway indicate a catabolic pattern. In fact, the reduced intrathymic levels of S1P kinase activity point out reduced a level of S1P along with infection.

Sphingosine-1-phosphate, via signaling through the S1PR1 receptor, has been previously shown to be a chemotactic factor for a variety of cell types including T lymphocytes [24], [43], [44]. The S1P receptors are expressed on diverse cell types and tissues, suggesting that the signaling pathway mediated by this receptor may be involved in multiple physiological processes [45], [46]. Several studies have demonstrated that the S1PR1 expression on T cells has a crucial function in the process by which differentiated thymocytes exit from the thymus and enter the blood. Under physiological conditions, the S1P1 receptor level is up-regulated in mature single positive thymocytes relative to immature double-positive thymocytes [24]. During T cell development, TCR signaling induces the expression of CD69, which downregulates S1PR1 surface expression, preventing the premature S1P-mediated thymocyte export from the thymus [25], [47], [48].

We found an increase in the expression of the S1PR1 and S1PR3 on CD4^−^CD8^−^ T cells during the acute phase of *T. cruzi* infection. This increased expression of both chemotatic attractant receptors involved in thymocyte trafficking could lead to high sensitization of the CD4^−^CD8^−^ T cells to the reduced levels of S1P in the infected thymus. In fact, these cells responded to lower levels of S1P and migrated *ex vivo*. In the light of these findings, and considering that the persistent use of agonists causes desensitization of the S1P pathway, we took advantage of the *in vivo* use of prolonged FTY720 treatment, which is known to lead to internalization and lysosomal degradation of S1P receptors [49]. Such process allows receptors to recycle, thus maintaining a functional receptor reserve, which in turn results in a blockage of the thymocyte emigration to the periphery [33], [50]. Other studies have used this S1P receptor inhibitor therapeutically in experimental murine *T. cruzi* infections and have shown a reduction of protective immunity due to sequestration of the lymphocytes in lymphoid organs, thus blocking their re-circulation mediated by sphingosine-1-phosphate receptors [51]. In our model, the onset of acquired immunity was not affected, since we started administrating FTY720 on day 6 post-infection, just before thymic atrophy got underway. Importantly, our i*n vivo* studies with FTY720 demonstrated the accumulation of double-negative thymocytes, suggesting that the thymic egress of CD4^−^CD8^−^ T cells during acute *T. cruzi* infection is rapidly regulated by the S1P receptors.

These findings indicate that chagasic acute infection promotes thymic alterations, which result in modulation of S1P-S1P1 receptor axes on intrathymic CD4^−^CD8^−^ T cells. This switch renders undifferentiating thymocytes capable of responding to chemotaxis mediated by S1P during thymic emigration. Indeed, CD4^−^CD8^−^ peripheral T cells, have been proposed to originate in the thymus by escaping negative selection followed by migration in the periphery [52]. The presence of immature CD4^−^CD8^−^ T cells bearing low TCR levels (DN3 and DN4) in the periphery may have implications for the host-parasite interplay, since they could be activated *ex vivo*. Previous findings revealed that unconventional CD4^−^CD8^−^ T cells in the periphery are able of recognizing antigens and subsequently signaling through their TCR in a MHC-independent manner [53], [54]. It has been demonstrated that the peripheral CD4^−^CD8^−^ T lymphocytes can undergo extrathymic T cell lymphopoiesis and that this process is severely compromised in IL-15^−/−^ and IL-2Rβ^−/−^ mice [55]. These cells have been shown to have pathogenetic properties in other models, in which their frequencies are increased. Several studies provided a direct link between CD4^−^CD8^−^ T cells and the development of human autoimmune diseases. In patients with systemic lupus erythematous, these cells are the major producers of IL-17 and infiltrate the kidneys [21]. CD4^−^CD8^−^ T cells have also been found in the blood of myasthenia gravis patients and normalization in the number of these cells was seen after thymectomy, in parallel with clinical improvement and reduction in anti-acetylcholine receptor antibody titers [56]. While both systemic lupus erythematous and myasthenia gravis are a primary autoimmune disease, in Chagas disease the autoimmune manifestations are considered to be secondary to the parasite-driven tissue damage [57]. Around 30% of *T. cruzi* chronically infected individuals are at risk of developing myocardial damage. The mechanisms underlying heart disease are linked to several processes like microvascular alteration, and molecular-mimicry related autoimmune reactions. Although different studies have demonstrated in autoimmune disease and infection models the presence of immature thymocytes in the periphery, the pathophisiological consequences of this phenomenon have not yet been elucidated. It is possible that premature released immature thymocyte could express forbidden αβTCR with self-antigen specificities and thus induce autoimmune responses.

Importantly, we showed that the purified thymic CD4^−^CD8^−^ T cells from *T. cruzi-*infected mice expressed high contents of TNF-α and IL-17a transcripts upon activation.

CD4^−^CD8^−^ T cells have been reported to play a key role in various models of infections. These lymphocytes are the major responding T cell subset in the lung of mice infected with *F. Tularensis bacterium*, releasing large amounts of IL-17a during the early stages of infection whereas CD4^+^ T cells start to produce this cytokine later [20]. As these cytokines mediate pro-inflammatory responses, the extrathymic CD4^−^CD8^−^ T cells may have a role in the pathogenesis of Chagas diseases. Most interestingly, we found that patients having the cardiac form of chronic human Chagas disease bear higher levels of circulating CD4^−^CD8^−^ T lymphocytes than non-infected individuals. These data suggest that this T cell subset might be associated with the development of the cardiac form of the disease.

Overall, the studies in the mouse model indicate an imbalance of the S1P metabolic pathway, leading to reduced levels of the bioactive natural agonist S1P in the thymus during the experimental *T. cruzi* acute infection. This imbalance was associated with an increased expression of the S1PR1 and S1PR3 receptors in CD4^−^CD8^−^ T cells. Moreover, migration of CD4^−^CD8^−^ T cells from infected thymus is influenced by S1P both *ex vivo* and *in vivo*, pointing out an important role of S1P receptors in regulating the abnormal release of immature thymocytes to the periphery in acute Chagas disease. It is possible that CD4^−^CD8^−^ T cells have a role in modulating adaptive immune responses in *T. cruzi* infections as we found they can induce activation of pro-inflammatory cytokine genes when they themselves are activated.

Lastly, our results indicate a connection between the changes in the levels of the CD4^−^CD8^−^ T cell subset and the severity of myocardial lesions in human Chagas disease. These observations identify a potential clinical marker of disease progression.

## Supporting Information

Figure S1 **Thymic atrophy in acute phase of** ***T. cruzi*** **infection.** Representative flow cytometric profiles showing CD4^+^, CD4^+^CD8^+^ and CD4^−^CD8^−^ thymic subpopulations during the course of infection. Of note, DP thymocytes virtually disappeared after 21 days post-infection.
(TIF)

Figure S2 **Intrathymic injection of FITC randomly stained the thymocyte subpolulations.** FACS plots showing a high proportion of thymocytes randomly stained by the intrathymic inoculation of FITC in both uninfected (upper panels) and infected mice (bottom panels).
(TIF)

Figure S3 ***Trypanosoma cruzi*** **acute infection promotes the release of FITC^+^ thymocyte CD4^−^CD8^−^CD44^−^CD25^−^ cells to the periphery.** Mice were intrathymically injected with FITC solution or PBS only as a control. After 24 h, spleens were harvested and cells stained with anti-CD4, anti-CD8, anti-CD44 and anti-CD25 for the identification of the specific subsets of CD4^−^CD8^−^ recent thymic emigrants (RTEs) by flow cytometry. (**A**) Upper panels represent the percentage of total FITC^+^ CD4^−^CD8^−^ T cells in spleen during *T. cruzi* acute infection and uninfected mice, while bottom panels represent the percentage of DN1 (CD44^+^CD25^−^), DN2 (CD44^+^CD25^+^), DN3 (CD44^−^CD25^+^) and DN4 (CD44^−^CD25^−^) among FITC^+^CD4^−^CD8^−^ migrating cells (**B**) Graphs represent the absolute number of splenic FITC^+^ DN thymocyte subsets regarding the expression of CD44 and CD25 markers during *T. cruzi* acute infection and uninfected mice. Results are expressed as mean ± SE (n = 3–5 mice per group). Differences between uninfected and infected mice are significant * (*p*<0.05), ** (*p*<0.01), *** (*p*<0.001).
(TIF)
