# Supplementary figures and images for: Early Double-Negative Thymocyte Export in Trypanosoma cruzi Infection Is Restricted by Sphingosine Receptors and Associated with Human Chagas Disease
Source: PLoS Negl Trop Dis. 2014 Oct 16;8(10):e3203. doi: 10.1371/journal.pntd.0003203 (PMC4199546; doi:10.1371/journal.pntd.0003203)

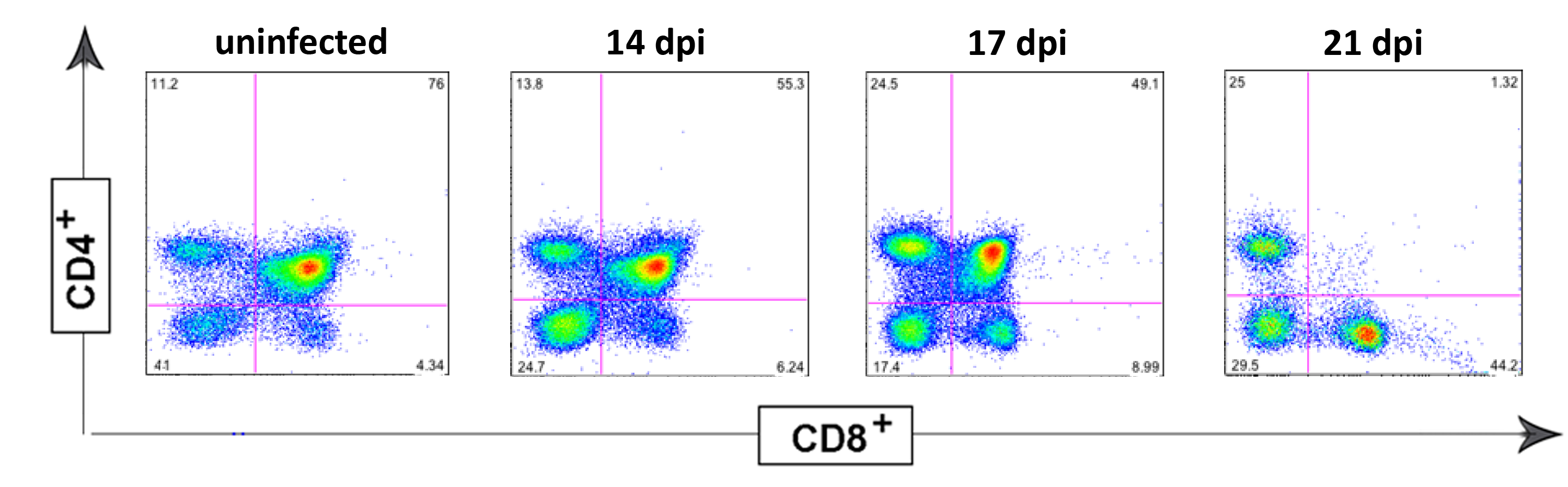

Supplement: Figure S1 — Thymic atrophy in acute phase of T. cruzi infection. Representative flow cytometric profiles showing CD4+, CD4+CD8+ and CD4−CD8− thymic subpopulations during the course of infection. Of note, DP thymocytes virtually disappeared after 21 days post-infection. (TIF) [file pntd.0003203.s001.tif]

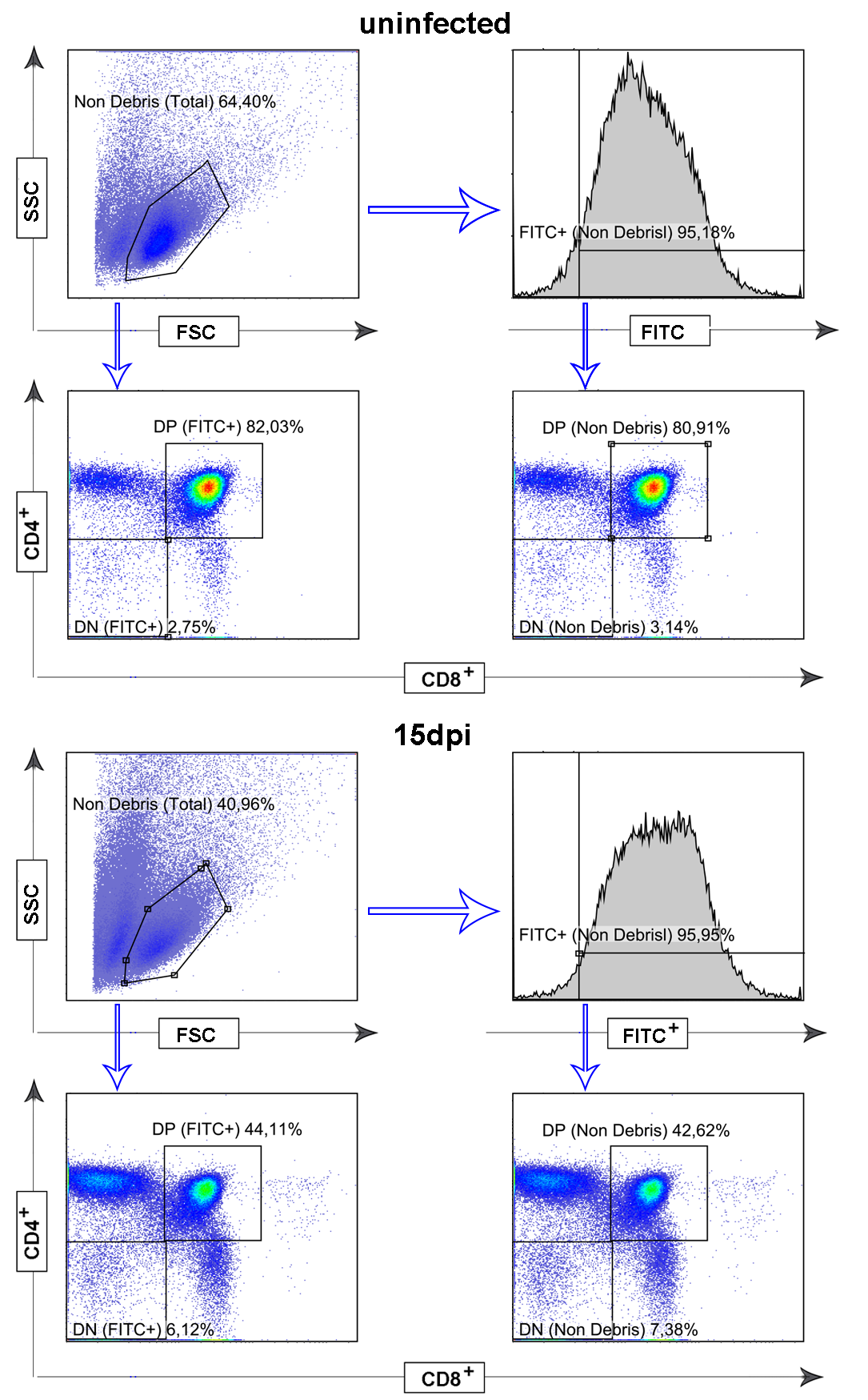

Supplement: Figure S2 — Intrathymic injection of FITC randomly stained the thymocyte subpolulations. FACS plots showing a high proportion of thymocytes randomly stained by the intrathymic inoculation of FITC in both uninfected (upper panels) and infected mice (bottom panels). (TIF) [file pntd.0003203.s002.tif]

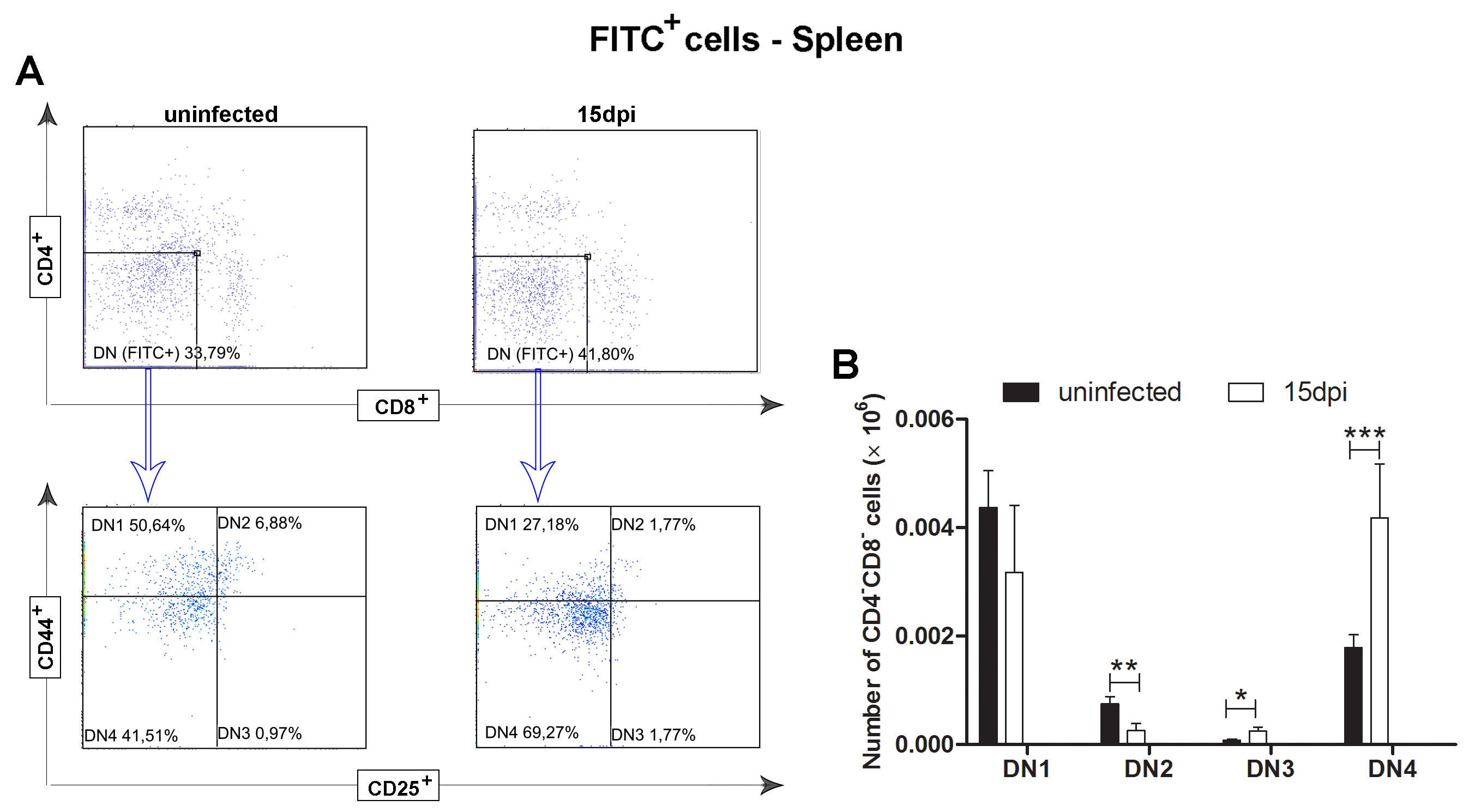

Supplement: Figure S3 — Trypanosoma cruzi acute infection promotes the release of FITC+ thymocyte CD4−CD8−CD44−CD25− cells to the periphery. Mice were intrathymically injected with FITC solution or PBS only as a control. After 24 h, spleens were harvested and cells stained with anti-CD4, anti-CD8, anti-CD44 and anti-CD25 for the identification of the specific subsets of CD4−CD8− recent thymic emigrants (RTEs) by flow cytometry. (A) Upper panels represent the percentage of total FITC+ CD4−CD8− T cells in spleen during T. cruzi acute infection and uninfected mice, while bottom panels represent the percentage of DN1 (CD44+CD25−), DN2 (CD44+CD25+), DN3 (CD44−CD25+) and DN4 (CD44−CD25−) among FITC+CD4−CD8− migrating cells (B) Graphs represent the absolute number of splenic FITC+ DN thymocyte subsets regarding the expression of CD44 and CD25 markers during T. cruzi acute infection and uninfected mice. Results are expressed as mean ± SE (n = 3–5 mice per group). Differences between uninfected and infected mice are significant * (p<0.05), ** (p<0.01), *** (p<0.001). (TIF) [file pntd.0003203.s003.tif]
